# Direct Measurement of Optical Force Induced by Near-Field Plasmonic Cavity Using Dynamic Mode AFM

**DOI:** 10.1038/srep16216

**Published:** 2015-11-20

**Authors:** Dongshi Guan, Zhi Hong Hang, Zsolt Marcet, Hui Liu, I. I. Kravchenko, C. T. Chan, H. B. Chan, Penger Tong

**Affiliations:** 1 Department of Physics, Hong Kong University of Science and Technology, Clear Water Bay, Kowloon, Hong Kong; 2 College of Physics, Optoelectronics, and Energy and Collaborative Innovation Center of Suzhou Nano Science and Technology, Soochow University, Suzhou 215006, China; 3 Department of Physics, University of Florida, Gainesville, Florida 32611, USA; 4 National Laboratory of Solid State Microstructures and Department of Physics, Collaborative Innovation Center of Advanced Microstructures, Nanjing University, Nanjing 210093, China; 5 Center for Nanophase Materials Sciences, Oak Ridge National Laboratory, Oak Ridge, Tennessee 37830, USA; 6 Institute for Advanced Study, Hong Kong University of Science and Technology, Clear Water Bay, Kowloon, Hong Kong; 7 William Mong Institute of Nano Science and Technology, Hong Kong University of Science and Technology, Clear Water Bay, Kowloon, Hong Kong

## Abstract

Plasmonic nanostructures have attracted much attention in recent years because of their potential applications in optical manipulation through near-field enhancement. Continuing experimental efforts have been made to develop accurate techniques to directly measure the near-field optical force induced by the plasmonic nanostructures in the visible frequency range. In this work, we report a new application of dynamic mode atomic force microscopy (DM-AFM) in the measurement of the enhanced optical force acting on a nano-structured plasmonic resonant cavity. The plasmonic cavity is made of an upper gold-coated glass sphere and a lower quartz substrate patterned with an array of subwavelength gold disks. In the near-field when the sphere is positioned close to the disk array, plasmonic resonance is excited in the cavity and the induced force by a 1550 nm infrared laser is found to be increased by an order of magnitude compared with the photon pressure generated by the same laser light. The experiment demonstrates that DM-AFM is a powerful tool for the study of light induced forces and their enhancement in plasmonic nanostructures.

Electromagnetic (EM) radiation has long been found to exert optical pressure on objects. A tightly focused laser beam can produce a strong light intensity gradient around the focal spot, which can be used to trap a small particle. This technique, more commonly called optical tweezers^1^,^2^, has emerged as a very useful tool to manipulate micro- or nano-meter sized particles and is widely used in physics^3^, chemistry^4^ and biology^5^. For a given laser beam, the maximal optical force (or pressure) that can be exerted to a particle is bound by the light intensity and laser wavelength or by the intensity gradient. In addition, the laser focal size is limited by diffraction. A more efficient way is needed to fully utilize the available photons and to boost the efficiency, which is essential for applications. Optical resonant cavities such as the whispering-gallery mode cavity and Fabry-Perot cavity, in which photons can bounce back and forth multiple times between the cavity walls, have been used as an efficient way to recycle photons^6^. Simple metallic surfaces are used in most of the conventional optical cavities.

Recently, surface plasmons (SPs) in nanostructures have attracted growing scientific and technological interests. Due to the strong local field enhancement, SPs find important applications in light confinement, nonlinear optics and optical sensors^7^. Since the advent of metamaterials^8^, the applications of SPs are also extended to some new areas, such as negative refraction^8^, super imaging^9^ and cloaking^10^. The design of nano-structured EM resonant elements expands our capabilities to have a better control of photons and light manipulation. Conventionally, the development of metamaterials focused mainly on the control of light propagation properties. However, it is well known that EM resonances can significantly enhance the local field intensity, which is directly connected to the induced optical force. Many efforts have been made recently to design new metamaterials to realize devices for optical force enhancement^11^,^12^,^13^,^14^. On the experimental side, continuing efforts have been made to identify accurate techniques to directly measure the near-field optical force induced by the plasmonic nanostructures in the visible frequency range^15^,^16^,^17^,^18^. The development of new experimental tools is essential for realizing the potential applications.

Atomic force microscopy (AFM), which measures the interaction force between an nano-scale cantilever tip and sample^19^,^20^, is optimized for high-resolution force measurement. It has been used to measure van der Waals forces^19^, Casimir forces^21^,^22^, single molecule stretching and rupture forces^23^,^24^, and optical forces^15^,^25^. It is an ideal tool for the high-precision measurement of the optical force induced by nano-structured metamaterials. AFM can be operated under either static or dynamic mode. In the dynamic mode (DM-AFM), an external oscillatory force is applied and information about the cantilever oscillation is monitored in addition to the static deflection^20^,^26^. As a sensitive force apparatus, DM-AFM can measure optical forces with a minimal amount of laser power and thus reduces unwanted effects associated with high laser power, such as nonlinear optical effects and strong photo-thermal effects.

In this paper, we report a new application of DM-AFM in the measurement of the enhanced optical force acting on a nano-structured plasmonic resonant cavity at near-infrared wavelength (*λ *= 1550 nm). As sketched in Fig. 1, the vertically oriented optical cavity under investigation has two parts. The upper surface of the cavity is made of a gold-coated glass sphere of radius *R* ≈ 15 μm, which is glued on the front end of a rectangular cantilever beam. The lower surface of the cavity is a flat quartz plate covered by a periodic array of subwavelength gold disks. An intensity-modulated laser beam enters the cavity from the bottom plate and exerts an oscillating optical force on the upper sphere. By using DM-AFM together with a phase-sensitive lock-in amplifier, we are able to observe a large enhancement of optical forces, when the cavity resonance is excited by an infrared laser of fixed wavelength 1550 nm with varying cavity separation *r* and gold disk size *d*. A good agreement is found between the experimental results and numerical simulations.

## Results

### Optical force measurement using DM-AFM

In the experiment, the DM-AFM is utilized to measure the optical force. The intensity modulated optical force *f*_*o*_(*t*) = *F*_*o*_ *cos*(*ω*′*t*), where *ω*′ is the modulation frequency of the laser intensity, causes the flexible cantilever beam to bend and the sphere’s vertical displacement *z*(*t*) (≡vertical deflection of the cantilever) is well described by the Langevin equation^27^,^28^,


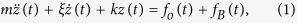


where *m* is the effective mass of the sphere-cantilever system, *ξż*(*t*) represents the drag force on the sphere with *ξ* being the friction coefficient, *kz*(*t*) is the elastic force resulting from the bending of the cantilever with a spring constant *k*, and *f*_*B*_(*t*) is the random Brownian force due to thermal fluctuations. While the mean value of *f*_*B*_(*t*) is zero, its autocorrelation function *C*(*τ*) has a form^29^, 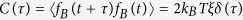, where *k*_*B*_*T* is the thermal energy of the system and *δ*(*τ*) is the *δ* function. One can analytically solve Eq. (1) in the frequency domain and obtain the power spectrum density (PSD)^30^,


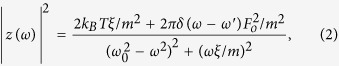


where *ω*_0_ = (*k/m*)^1/2^ is the mechanical resonant frequency of the sphere-cantilever system, and *ω *= 2π*f* is the angular frequency arriving from the Fourier transform of the measured time-varying cantilever signal. Note that the frequency *ω* is a variable in Eq. (2), which is different from the (fixed) laser modulation frequency *ω*′.

Figure 2 shows the measured PSD *|z*(*ω*)*|*^2^ as a function of *ω*, which is obtained with an optical cavity when the closest distance *r* between the surface of the sphere and the surface of the gold disks is equal to 1 μm. Hereafter, we refer to *r* as the cavity separation. The measured *|z*(*ω*)*|*^2^ shows a resonant peak centered at *ω*_0_* *= 43.8 kHz together with a sharp spike resulting from the optical force acting on the gold-coated glass sphere, which is intensity modulated at the driving frequency *ω*′ = 56.51 kHz (*f* ′* *= 9 kHz). The solid line is a fit to Eq. (2) with *m *= 3.46 × 10^−8^ g, *ω*_0_* *= 43.79 kHz, and *ξ *= 4.64 × 10^−8^ Ns/m. The experimental result agrees well with Eq. (2), confirming that the motion of the cantilever system can indeed be described by Eq. (1).

By filtering out the Brownian noise *f*_*B*_(*t*), one can also use a lock-in amplifier to directly measure the driven harmonic oscillator signal from Eq. (1) in the time domain^20^,^26^, *z*(*t*)* *= *Acos*(*ω*′*t+φ*), where the displacement amplitude *A* and phase delay *φ* are the two measurable quantities in the experiment. The force amplitude *F*_*o*_ can be calculated via


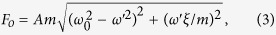


where *m*, *ω*_0_ and *ξ* can be measured by the PSD. Note that because of the fluid (air) dynamic interactions in the cavity gap, *ξ* will increase with decreasing *r* when *r* becomes small^30^. Therefore, it is important to accurately measure the PSD as a function of *r* in the near field region and use Eq. (3) to obtain the force amplitude *F*_*o*_.

The smallest force measurable by DM-AFM is limited by the measured PSD and frequency band width Δ*B* of the system^31^. From Eq. (2), we find


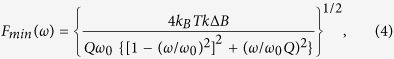


where Δ*B *= 7.8125 Hz is the bandwidth set by the lock-in amplifier, and *Q* =  *ω*_0_*m/ξ* (≈33) is the quality factor of the cantilever system. Using Eq. (4) and the fitting parameters obtained from Fig. 2, we find the force sensitivity of our DM-AFM is *F*_*min*_ ≈ 70 fN. In the experiment, we measure the optical force at the level of ~2 pN (see Section on Near-field optical force enhancement for more details) with integration time of 0.1 second and we can readily obtain force signals when the input laser power is reduced by a factor of 10 (down to 0.1 mW). Therefore, the actual sensitivity of our force measurement is better than 0.2 pN, which is not very far from the above theoretical estimate of ~0.1 pN. Under the same condition, we find the sensitivity of our static mode AFM is at the level of ~16 pN. Therefore, the sensitivity of the DM-AFM is improved by a factor of 80.

### Plasmonic resonant cavity and simulation results

Previous theoretical studies^12^,^13^ have shown that the cavity resonance occurs when two separate conditions on disk size *d* and the cavity separation *r* are satisfied simultaneously. In analogy to a half wavelength dipolar antenna, the single-disk plasmonic resonance can be excited when *d* is slightly smaller than half a wavelength. At the same time, the value of *r* has to be small enough in order to have a strong coupling with high EM energy density stored inside the cavity. On the other hand, *r* cannot be too small, as it will reduce the EM energy stored inside the cavity or even short the circuit. Typically, the value of *r* at the resonance is in the range of tens of nanometers^12^,^13^. These two parameters are varied separately in the experiment.

We carry out a numerical calculation of the induced electric and magnetic field distributions inside a plasmonic resonant cavity used in the experiment, as shown in Fig. 3(a,b). A plane wave of wavelength *λ *= 1550 nm is incident from the bottom quartz substrate with *E*_*y*_ polarization. A very strong *E*_*z*_ field of opposite sign is found at two edges of the nano-disk, indicating that a strong oscillating electric dipole is formed across the nano-disk. This oscillating electric dipole together with its image dipole formed in the upper gold plate give rise to an electric attraction between the nano-disk and gold plate when the cavity separation *r* is small enough. On the other hand, the magnetic field is concentrated at the center of the cavity, resulting from the antiparallel currents between the nano-disk and gold plate and a repulsive force is induced. However, the enhancement of the magnetic field is smaller than that of the electric field due to the field penetration and materials loss in gold. Thus a net attractive force is expected^12^. Both the electric and magnetic field amplitudes in the cavity are enhanced by a factor more than 10 times compared with the maximal value of the non-resonance field in the same cavity.

Along with the enhanced electric and magnetic fields in the cavity, a strong EM energy *U* is stored inside the cavity. For a high-quality-factor resonance cavity of small separation *r*, the enhanced optical force acting on the cavity wall is –*dU/dr*. Similar to the experiment to be described below, we fix the laser wavelength at *λ* = 1550 nm and vary *r* and *d* in the simulation, and search for an optimal configuration to have the largest enhancement factor *E* of the induced optical force acting on the top gold plate relative to its non-resonance value. As shown in Fig. 3(c), a strong resonance is found at *r *= 30 nm for the cavity with gold disk size *d *= 380 nm. The calculated value of *E* starts to increase rapidly when the separation *r* becomes smaller than ~100 nm and reaches its peak value of *E* ≈ 50 at *r *= 30 ±5 nm. Similar enhancement of optical force was predicted for a similar setup^12^.

In the actual experiment, instead of using a single nano-disk which is difficult to align with the cantilever experimentally, we use a periodic array of nano-disks together with a large gold-coated glass sphere on the top, forming *N* parallel nano-disk cavities. As a result, the total force measured on the top sphere will be a sum of the force induced by *N* individual nano-disk cavities, assuming that the lateral coupling between neighboring nano-disks is negligible. Indeed, Fig. 3 reveals that the electric and magnetic fields in the cavity decay rapidly and their absolute value at a distance 100 nm away from the nano-disk is minimal. Based on this simulation, we design the periodic array of nano-disks with a 200 nm separation between the nano-disks, ensuring that the overlap of the electric and magnetic fields from the neighboring nano-disks is negligibly small in the wavelength range we are interested. In the experiment, a typical value of *N* is ~100 for the nano-disk array with *d *= 625 nm.

### Measured optical force in non-resonant cavities

Figure 4(a,b) show, respectively, the measured displacement amplitude *A* and phase delay *φ* as a function of the cavity separation *r*. They are measured with an infrared laser when the lower surface of the cavity is replaced by a plain quartz plate without any gold disk on it. It is seen that the values of *A* and *φ* remain approximately constant with varying values of *r*, except when the glass sphere touches the quartz substrate (*r* ~ 0). In this configuration, the infrared laser beam goes though the unpatterned quartz plate and directly hits on the gold-coated sphere, and the measured force *F*_*o*_ is simply the conventional optical pressure on the gold-coated sphere. The value of *F*_*o*_ is proportional to the laser intensity and has no dependence on *r*. Figure 4(a,b) thus reveal the typical behavior of *A* (or *F*_*o*_) and *φ* in a non-resonant cavity.

As mentioned above, the plasmonic resonant cavity with a periodic array of gold disks is designed for an infrared light with wavelength *λ *= 1550 nm. When a laser light with a wavelength outside the designed wavelength range illuminates the cavity, one expects no plasmonic resonance effect. Figure 4(c,d) show, respectively, the measured *A* and *φ* as a function of *r*, when an alignment red laser of wavelength *λ *= 635 nm incident into an array of gold disks. By varying the gold disk diameter *d* from 300 nm to 750 nm, we find that the measured *A* and *φ* remain constant and no resonance effect is found as expected.

### Measured optical force in plasmonic resonant cavities

Figure 4(e,f) show, respectively, the repeated measurements of *A*(*r*) and *φ*(*r*) for the same array of gold disks as those used in Fig. 4(c,d), but with the alignment red laser being replaced by the infrared laser of wavelength *λ* = 1550 nm. Figure 4(g,h) show the magnified plots of the measured *A*(*r*) and *φ*(*r*) shown in Fig. 4(e,f) in the near-filed region. Compared to Fig. 4(c,d), the measured *A*(*r*) and *φ*(*r*) reveal several interesting new features. (i) In the far field with the separation |*r*| > 3 μm, the measured *A* for different values of *d* varies considerably. This is because the transmission coefficient *T*(*d*) of the patterned substrate has a strong dependence on the gold disk diameter *d* (see more discussions below). As a result, the measured *A*, which is proportional to the laser intensity incident on the gold-coated glass sphere, changes with *d*. (ii) With *d* in the range of 600-700 nm, the measured *A*(*r*) oscillates with *r* and the oscillation period is in multiples of half a wavelength. This behavior indicates that the laser beam is reflected back and forth between the gold-coated sphere and the gold disk array that forms a Fabry-Perot cavity. (iii) In the near field with the cavity separation |*r*| < 0.5 μm and with *d* in the range of 625 ± 25 nm, the value of *A* increases rapidly as |*r*| descreases. This effect suggests that the local light intensity near the gold disks with *d* in the range of 625 ± 25 nm is greatly enhanced, which is a resonant response of the plasmonic cavity. For the cavities with *d* in the range of 250–500 nm (some of the curves are not shown for clarity), they are off resonance with respect to the infrared laser used. Those cavities with intermediate ranges of *d* (*d* < 600 nm and *d* > 650 nm) are at different levels of partial resonance, depending on how close they are to the resonance condition. (iv) The phase difference between the modulated laser and cantilever mechanical response *φ*(*r*) also varies with *d*. To further understand the resonant behavior of the measured *A*(*r*) and *φ*(*r*), we now analyze the far-field and near-filed responses separately.

### Far-field optical force and optical transmission of patterned substrate

To examine the effect of the optical transmission of the patterned substrate on the optical force, we measure the transmission coefficient *T*(*d*) for different patterned substrates using the standard method of Fourier transform infrared spectroscopy (FTIR)^41^. Figure 5 shows the measured *T*(*d*) (red circles) as a function of *d* for the infrared light of wavelength *λ *= 1550 nm. To compare the results with the DM-AFM measurements, we compute the normalized displacement amplitude *A*′_*1550*_(*d*)* *= *A*(*d*)*/A*_0_ (black solid squares) at *r *= 4.8 μm with *A*_0_ being the displacement amplitude obtained using the non-resonant cavity as shown in Fig 4(a). It is seen that the measured *A*′_*1550*_(*d*) by DM-AFM agrees well with the measured *T*(*d*) by FTIR. The minimum value of *A*′_*1550*_(*d*) is ~0.16 at *d *= 700 nm, which agrees with the numerical calculation of the transmission coefficient (green diamonds). Figure 5 thus confirms that the changes of the measured *A* in the far field are indeed caused by the variation of *T*(*d*) with *d*. The transmission minimum is caused by the excitation of the plasmonic dipole mode of the gold disks, whose radius (*d *= 700 nm) is slightly smaller than half the wavelength (*λ*/2* *= 775 nm).

For comparison, we also plot, in Fig. 5, the normalized vibration amplitude *A*′_635_(*d*) (blue solid triangles) obtained in the same cavity, but with the incident laser being replaced by the alignment red laser of wavelength *λ *= 635 nm. As mentioned, the red laser does not excite the plasmonic dipoles of the gold disks. As a result, the measured *A*′_635_(*d*) is high (≈0.92) and remains unchanged with *d* as expected. This result also confirms that the variations of the measured *A*′_*1550*_(*d*) [and *T*(*d*)] are not caused by the change in area fraction occupied by the periodic array of gold disks on the substrate. Rather, they result from the resonant excitation of plasmonic waves on the patterned surface.

### Far-field phase delay and thermal effect

While we have used low laser power (~1 mW) to reduce the thermal effect, it nevertheless has an effect measurable by DM-AFM. The long rectangular cantilever beam is extremely sensitive to slight temperature variations either in the environment or resulting from direct absorption of laser light, which generate non-uniform thermal expansion and bending along the beam^25^,^32^,^33^. While the thermally induced bending is improved by coating a thin layer of gold on the cantilever to increase its reflectivity and hence reduce direct absorption of laser light (see details in the Methods section), there is still a residual thermal response to the modulated laser light. As a result, the cantilever beam has an oscillatory bending, as if there is an “AC thermal force” acting on the cantilever in addition to the optical force. As a general AC response, the thermally induced oscillatory bending can be phenomenologically modeled as 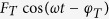, with two free parameters; one is the amplitude of the “thermal force” *F*_*T*_ and the other is the phase delay *φ*_*T*_ between the induced thermal force and the optical driving force to be measured. The total force *F* acting on the cantilever then can be written as, 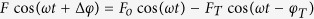, which is shown graphically in the inset of Fig. 6. This equation can be rewritten in the complex form,


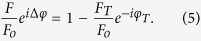


In the above, *F*_*o*_ is the amplitude of the optical force and the minus sign is used for convenience, as *F*_*T*_ can be either positive or negative.

There are five parameters in Eq. (5), among which *F* is measured for different values of *d* and *F*_*o*_ in the far-field is proportional to the measured *T*(*d*) as shown in Fig. 5. From our numerical simulations, we find that heat is generated mostly by the lower patterned plate, as its absorption (~8%) is much larger than that of the upper gold layer (<1%). Furthermore, the optical absorption of the bottom nano-structured substrate is found to be approximately the same for all gold disk sizes. The observed transmission variations as shown in Fig. 5 are caused mainly by the reflection of the nano-structured substrate with varying disk size *d*. We hence assume *F*_*T*_ is a constant independent of *d*. To estimate the value of *φ*_*T*_, we assume that the phase delay is caused primarily by the transfer of modulated laser heating from the lower patterned plate, which has the largest adsorption, to the upper cantilever beam. With the geometry of the resonant cavity and an estimated temperature difference, we find the Rayleigh number of the system is very small (*Ra* ≈ 2.5 × 10^−5^), indicating that convection of air inside the cavity is negligibly small and heat is transported primarily by conduction^34^. As a result, the phase delay *φ*_*T*_ can be written as *φ*_*T*_ ≈ *ωτ*_0,_ where *τ*_0_ is the thermal diffusion time in air from the lower patterned surface to the upper gold-coated cantilever beam. Given the geometry of our setup, we find *φ*_*T*_ ≈ 64.8^o^ at *r *= 5 μm. With the fixed value of *φ*_*T*_, one can calculate Δ*φ* (and *F*/*F*_*o*_) using Eq. (5) with only one fitting parameter, *F*_*T*_/*F*_*o*_* *= *α*/*T*(*d*), for different values of *d*, and compare the results with the directly measured Δ*φ*.

Figure 6 shows the measured phase shift Δ*φ *= *φ*(*d*) – *φ*(*d *= 250 nm) as a function of *d*, where *φ*(*d *= 250 nm) is the non-resonant reference phase obtained with *d *= 250 nm. The red circles are the calculated Δ*φ* using the dual force model in Eq. (5) with a constant value of *α *= 0.17. With the same fitting parameter *α*, we are able to reproduce the main feature of the measured Δ*φ* in the far-field (red circles vs. black squares in Fig. 6). Figure 6 thus demonstrates that our assumption about *φ*_*T*_ is adequate at the phenomenological level to describe the data in the far-field region (*r* > 3 μm). In Table 1, we list the obtained values of *F*_*o*_*/F*_*T*_ and *F*_*o*_*/F* for different disk patterns. It is seen that while the value of *F*_*o*_*/F*_*T*_ varies from 5 to 1 for different values of *d*, the obtained values of *F*_*o*_*/F* remain close to unity, indicating that the measured total force *F* can be approximately treated as the optical force for simplicity. The main consequence of the thermal effect in our system is to introduce a phase delay in the AC response of the cantilever without much influence to the measured amplitude of the optical force.

### Near-field optical force enhancement

Figure 7 shows the optical force *F*′ normalized by transmission as a function of cavity separation *r,* which is obtained in the plasmonic resonant cavity with different gold disk diameters *d*. Here *F*′ is defined as *F*′* *= *F*_*o*_*T*(*625*)*/T*(*d*), where *F*_*o*_ is obtained from the measured vibration amplitude *A* using Eq. (3) with the parameters *m*, *ω*_0_, and *ξ* obtained from the measured PSD *|z*(*ω*)*|*^2^. The values of *F*_*o*_ are normalized by the ratio *T*(*d*)/*T*(625), where *T*(625) is the measured transmission coefficient with *d *= 625 nm, at which the measured *F*_*o*_ shows the largest enhancement. In the far-field region (*r* > 3 μm), there is no cavity resonance and the gold-coated glass sphere far away from the nano-disk array only feels the incoming light from the other wall of the cavity, i.e., the transmitted light through the nano-disk array. In this case, the optical force acting on the glass sphere results only from the conventional optical pressure, which is proportion to the transmitted light into the cavity. As shown in Fig. 7, all the optical forces become equal for *r* > 3 μm after the transmission normalization. In the near-field region (*r *< 0.5 μm), however, the cavity resonance becomes more pronounced. In this case, the measured *F*′ is no longer proportional to the transmitted light and shows strong variations with both *r* and *d*, a signature of cavity resonance which is clearly revealed in Fig. 7. It is seen that for *d *= 625 nm, the strongest coupling and hence the largest optical force are achieved at *r *= 30 nm.

In the experiment, a laser beam of diameter ~190 μm is used to ensure that for each measurement the sphere is readily aligned to the center of the Gaussian laser beam and the focused beam does not diverge over the vertical moving distance of ~5 μm. As a result, a portion of the laser beam is incident directly on the AFM cantilever without going through the resonant cavity. This portion of the laser beam applies an optical pressure to the cantilever, providing a background optical force to the measured *F*′, which is independent of *r*. To further examine this effect, we carry out a comparative optical force measurement with the gold-coated glass sphere replaced by an uncoated glass sphere of the same size. Because the uncoated glass sphere is transparent, the system exhibits no resonance effect. In this way, one only measures the background force acting on the gold-coated cantilever beam. The black solid circles (with a dashed line) in Fig. 7 show the measured optical force for the uncoated glass sphere, which has a constant mean value (=1.95 pN) independent of cavity separation *r*. No force enhancement is observed in the system, further confirming that the optical force enhancement as shown in Fig. 7 is indeed produced by the patterned plasmonic resonant cavity. For the optical cavities with *d *= 300 nm and *d *= 500 nm, because they are off resonance, the resulting values of *F*′ remain invariant with *r* and are slightly larger than the background force as expected. By removing the *r*-independent background force from the measured *F*′, we find the enhancement factor *E* under the experimental resonant conditions with *λ *= 1550 nm, *d *= 625 nm and *r *= 30 nm is *E *= (7.90–1.95)/(2.28–1.95)* *= 18, relative to the non-resonant value.

## Discussions

By comparing Figs 7 and 3(c), we find that the measured *F*′(*r*) for *d *= 625 nm follows the same trend as that of the simulation curve and in both cases, the maximal enhancement of the induced optical force occurs at *r *= 30 nm. The obtained resonant peak in the experiment, however, is broader and the peak height (i.e., the enhancement factor *E*) is smaller compared with the simulation values. In the numerical simulations, a parallel semi-infinite gold plate was used as the upper surface of the resonant cavity. In the actual experimental setup, a gold-coated glass sphere was used instead for the ease of optical alignment. In this case, the observed optical force enhancement can be viewed as an average over *N* (~100) parallel cavities. However, only a fraction of the gold disks form high-fidelity resonance with the upper gold-coated sphere and thus a less-pronounced enhancement factor *E* is observed. The cavity separation has a strong influence on the field leakage and the enhancement factor. In simulations, we find that the size of nano-disk at resonance is shifted from ~700 nm (for a single nano-disk) to ~380 nm (for a coupled plasmonic cavity at proximity *r *= 30 nm) under the illumination of a 1550 nm laser. As most of the parallel cavities in the experiment are with a separation ~100 nm, the coupling is weakened and thus the size of the nano-disks that we observed with the largest enhancement factor *E* is only reduced modestly from ~700 nm for a single nano-disk resonance to the actual gold disk size *đ *= 567.5 nm (see Table 1 for *d *= 625 nm). Even though our experimental setup is based on a curved surface of a sphere rather than an ideally flat supper surface, we are still able to achieve optical force enhancement in the nano-structured plasmonic cavities with the same set of values of *r* and *d*, as predicted by the numerical simulations.

Previously, optical tweezers^18^ and total internal reflection microscopy (TIRM)^35^,^36^ have been used to study the resonant response of plasmonic structures. Compared to these optical methods, our setup and technique have several important advantages. First, by a precise control of the AFM cantilever position, we are able to study the resonant phenomena in an optical cavity with cavity separation *r* varied from micro- to nano-meters, while it is difficult for TIRM to be operated at the length scales below 100 nm or above half a wavelength. Second, by using the dynamic mode AFM and lock-in technique, we can directly measure the optical force at the plasmonic resonance with high sensitivity on the order of 0.1 pN (with integration time of 0.1 s) in room temperature and standard atmospheric pressure. The near-field force measurement by the optical tweezers, on the other hand, will be significantly influenced by various near wall effects, such as light reflection and blockage, and the induced surface plasmonic waves by the optical tweezers themselves when they are placed very close to a metallic surface^18^. This is particularly true at the resonance.

The main challenge for the present technique is the near-field thermal effect on the AFM cantilever due to the modulated laser beam used, which is already set at a minimal level of intensity. It is noted that the force exerted by the EM radiation always has a direct electromagnetic force component and an indirect thermal effect. The thermal effect as discussed above becomes even more complicated in the near field^37^,^38^. At this moment, the direct electromagnetic force can be computed with high precision using numerical techniques, such as the Maxwell Stress Tensor approach. It would be highly desirable if the thermal effects can be calculated from first principles to the same level of accuracy. It is extremely challenging, however, as the near-field radiative heat transfer alone is already very complicated^39^,^40^, not to mention the additional complications resulting from other heat transfer pathways that are configuration and material dependent. More efforts are needed in order to understand the near-field thermal effects.

In summary, we have carried out a systematic study of optical forces in a plasmonic resonant cavity using the dynamic mode AFM. The plasmonic cavity is composed of an upper gold-coated glass sphere and a lower quartz substrate patterned with a periodic array of subwavelength gold disks. The measured optical force is found to have a strong dependence on the cavity separation *r* and the diameter *d* of the gold disks. The conventional optical force is obtained in the far-field (*r* > 3 μm) for different values of *d* and agrees well with the measured optical transmission. In the near-field (*r* < 0.5 μm), plasmonic resonance is excited in the cavity and the induced force by an infrared laser is increased by an order of magnitude compared with the usual optical pressure generated by the same laser light. The experiment demonstrates that dynamic mode AFM is a powerful tool for the study of EM-induced forces in plasmonic nanostructures.

## Methods

### Theoretical model and simulation

Previous theoretical studies^12^,^13^ have shown that there exists an antisymmetric mode in this plasmonic cavity, in which two antiparallel resonant currents oscillate along the top and bottom gold nano-disks. The induced antiparallel currents give rise to a repulsive force between the two nano-disks, whereas the accumulated opposite charges on the opposing edges of the two nano-disks lead to an attractive force. The net time-averaged force acting on the cavity is a result of competition between the two opposing electric and magnetic effects. In a recent microwave experiment^17^, a net repulsive force was observed, as the finite-size edge effect weakens the electric attraction. Unlike in the microwave regime, in the present experiment the optical loss of gold is not negligible and will reduce the current-induced repulsion significantly. As a result, a net attractive force is expected for the plasmonic resonant cavity^12^.

To further verify the above theoretical ideas in the present experiment, we carry out Finite-Difference-Time-Domain (Lumerical FDTD Solutions) simulations of the induced electric and magnetic field distributions inside a plasmonic resonant cavity used in the experiment. The time-averaged Maxwell stress tensor algorithm is then used to calculate the light induced force. As the diameter of the glass sphere is much larger than the size of the nano-disk and the thickness (~100 nm) of the gold layer coated on the glass sphere is much larger than the skin depth of gold for the infrared light (*λ *= 1550 nm), in simulations we simplify the top gold-coated glass sphere to a flat gold plate of semi-infinite thickness. Periodic boundary condition is applied on both the *x* and *y* directions and the size of unit cell is 200 nm larger than the disk diameter *d*. The numerical results are shown in Fig. 3.

### Assembly of plasmonic resonant cavity

**Figure 1:**
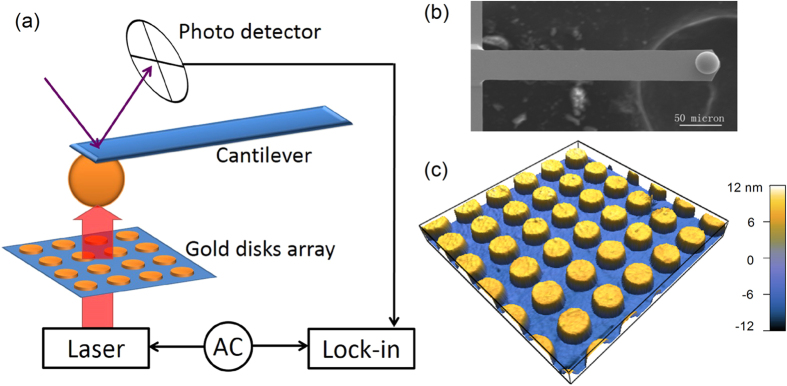
(**a**) Sketch of the experimental setup of the optical cavity and the dynamic mode AFM. (**b**) A scanning electron microscope (SEM) image showing the bottom view of a rectangular cantilever with a gold-coated glass sphere glued on its front end. (**c**) An AFM image showing a periodic array of gold disks of diameter *d* = 650 nm on a quartz substrate. The viewing area is 5 × 5 μm^2^.

**Figure 2:**
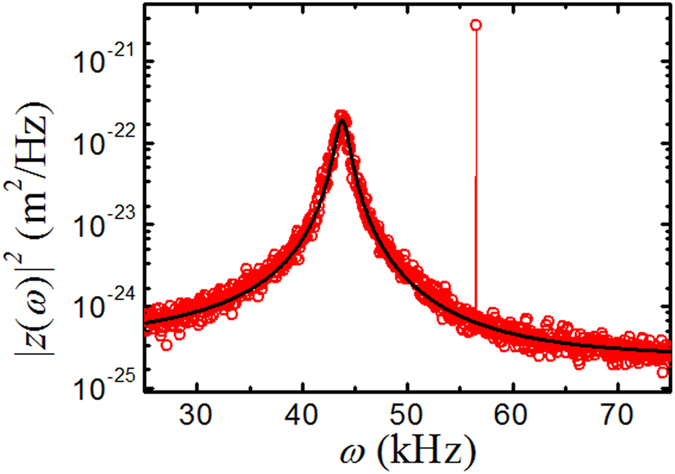
Measured power spectrum *|z*(*ω*)*|*^2^ as a function of angular frequency *ω*. The measurement is made with a plasmonic resonant cavity with a periodic array of gold disks of diameter *d* = 300 nm. The cavity separation is *r* = 1 μm and the intensity modulation frequency is *ω*′ = 56.51 kHz (*f* ′ = 9 kHz). The solid line is a fit to Eq. (2) with *m* = 3.46 × 10^−8^ g, *ω*_0_ = 43.79 kHz, and *ξ* = 4.64 × 10^−8^ Ns/m.

**Figure 3:**
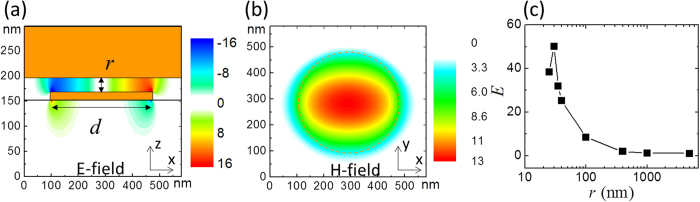
Calculated (a) z-component of the electric field distribution (side view of a mid-vertical-plane cut) and (b) x-component of the magnetic field distribution across the horizontal plane in the middle of the gap between the gold nano-disk and gold plate. A plane wave infrared laser light of *λ* = 1550 nm is incident to the plasmonic cavity from the bottom quartz substrate. The diameter of the nano-disk is *d *= 380 nm, its height is *τ *= 16 nm, and the cavity separation is set at *r *= 30 nm. The unit cell is 580 nm. The color bar indicates the field strength normalized by the maximum field strength obtained when a laser light of *λ *= 635 nm is incident to the non-resonant cavity. (**c**) Calculated enhancement factor *E* of the induced optical force acting on the top gold plate relative to its non-resonance value as a function of separation *r* for the cavity with gold disk size *d *= 380 nm. The solid line is drawn to guide the eye.

Figure 1(b) shows the bottom view of a rectangular cantilever beam with a gold-coated glass sphere glued on its front end. Commercial glass spheres (Microspheres-Nanospheres) of radius *R* ≈ 15 μm are used in the experiment. The AFM cantilevers used are silicon tipless micro-cantilevers (MikroMasch) with a spring constant *k *= 0.08 N/m. The assembly of the glass sphere to the cantilever is carried out using a motorized micromanipulator system together with a high-magnification stereo-microscope (Leica MZ16). The glass sphere is glued to the cantilever using a UV curable glue (Norland, NOA 81). When the glue is cured, the modified cantilever is cleaned with repeated rinses of isopropanol followed by a 20-minute plasma cleaning (Harrick Plasma PDC-32G). Finally, the cantilever with the glass sphere is coated with a 100 nm thick gold film using a vacuum sputter (Denton Explorer 14). This 100-nm-thick gold film is thick enough to make sure that the absorption of the system is determined by the gold film, which is much smaller than that of the highly n-doped silicon cantilever. At the same time, it is not too thick to substantially affect the intrinsic mechanical property of the cantilever. Such an optimization of the thickness of the gold film is essential to reduce the unwanted thermal uneven bending of the long rectangular cantilever beam.

Figure 1(c) shows an AFM image of a periodic array of gold disks of diameter *d *= 650 nm coated on a quartz substrate. The gold disk array is fabricated using electron beam lithography method followed by metal lift-off. In the experiment, we use different disk arrays with *d* varied in the range of 250-750 nm. The nominal values of *d* and the actual AFM-measured gold disk dimension are given in Table 1. The plasmonic resonant cavity is formed between the upper gold surface of the glass sphere and the lower gold disk patterned quartz plate. The cavity separation *r* is defined as the closest distance between the surface of the glass sphere and the surface of the gold disks. The value of *r* can be continuously varied using the z-axis piezo of the AFM. In the experiment, we change the lower plate of the resonant cavity by positioning the sphere over different disk arrays. For comparison, a plain quartz plate without any gold disk on it is also used to form a cavity that does not exhibit any resonance effects.

### Operation of DM-AFM

In the experiment, an AFM (MFP-3D, Asylum Research Inc.) is used for the optical force measurement. The AFM is operated under two modes. One is the thermal power spectral density (PSD) mode, which is used to measure the power spectrum, *|z*(*ω*)*|*^2^ (or equivalently *|z*(*f*)*|*^2^), of vertical deflections of the cantilever. Under this mode, the cantilever is positioned stationary. The voltage signal from the position-sensitive photo-detector [see Fig. 1(a)] is digitized at the sampling rate of 5 MHz. Typically, *|z*(*ω*)*|*^2^ is taken with a frequency resolution of 9.54 Hz and the averaging time for each *|z*(*ω*)*|*^2^ is kept at a fixed period of 5 minutes. The other mode is the dynamic mode AFM in which the lock-in technique is used to measure the optical force at a modulation frequency and the background noise is filtered out, removing the unwanted effects from other surface forces.

Figure 1(a) shows the basic setup of the dynamic mode AFM. An infrared laser of wavelength *λ *= 1550 nm is used in the experiment, and its intensity is modulated in the frequency range of 4-10 kHz. The intensity-modulated laser beam is vertically incident into the optical cavity from below through the patterned substrate. A collinear red laser of wavelength *λ *= 635 nm is used to align the optical setup. The AFM has a built-in optical access and a CCD camera, allowing us to directly observe the glass sphere glued on the cantilever, the gold disk pattern, and the red laser spot inside the optical cavity. This visualization system helps us to align the incident laser beam and move the glass sphere to the center of the laser spot. There is an optical filter placed in front of the AFM photodetector, which blocks the incident laser light at *λ *= 1550 nm. The transmittance of the optical filter is below 10^−5^ at *λ *= 1550 nm, ensuring that the IR light cannot enter the AFM detecting system. The laser modulation frequency is set at *ω*′* *= 56.51 kHz, at which we find the phase delay is *φ* = −170^o^ relative to the set value of *φ *= −90^o^ at resonance. This frequency is chosen to be close to the resonant frequency of the cantilever system (*ω*_0_ = 43.79 kHz), so that a measurable ac signal is obtained with a low laser intensity (~1 mW). In addition, the high modulation frequency helps to reduce the laser heating effect^32^,^33^.

All the AFM measurements are conducted in a lab room with a vibration-isolation floor. In addition, the entire optical cavity and AFM setup sits on an active vibration-isolation table, which is placed in an acoustic isolation hood (BCH-45, Asylum Research Inc.), further reducing the effect of surrounding vibrations. There is an air temperature controller (ATC) inside the acoustic hood, which keeps the hood temperature near the instrument at a constant value of 28 ± 0.1 ^o^C. In the experiment, we use the lowest possible intensity of the infrared laser, in order to minimize the local heating effect. The highest laser intensity used is 1.55 mW. Prior to each measurement, we turn on the whole AFM system and ATC and wait for at least 4 hours to make sure that the entire system reaches an equilibrium with the surroundings to minimize the thermal drift in the measurement system. At equilibrium, we find that the drift of the cavity separation *r* is about 1 nm/min. To avoid possible electrostatic interactions, a strip of polonium source (Staticmaster) is placed near the patterned substrate to neutralize its surface charges. In addition, the gold-coated sphere is electrically grounded.

### Data analysis

**Figure 4:**
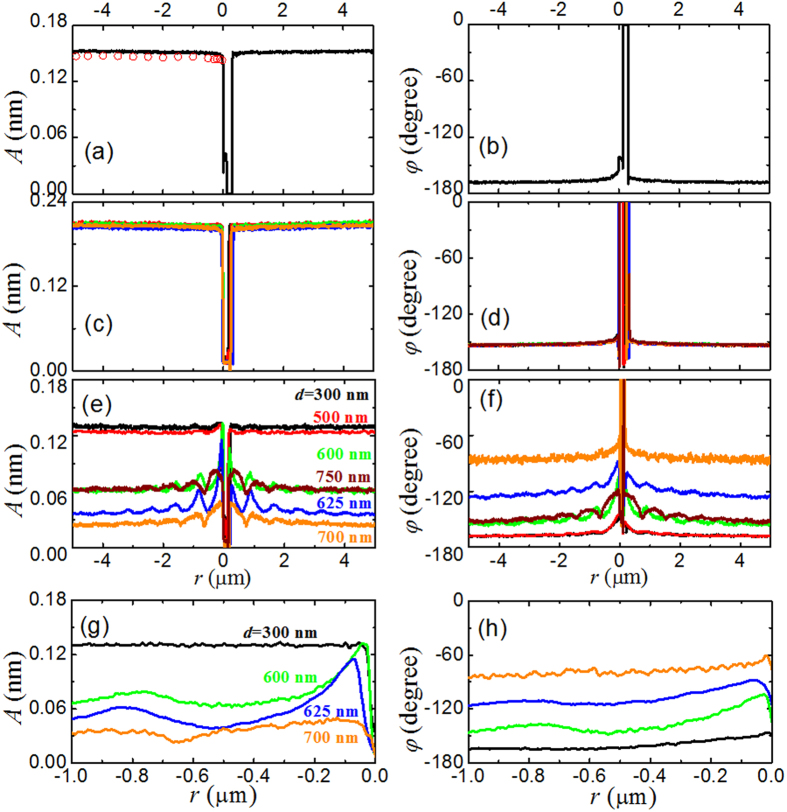
Measured displacement amplitude *A* and phase delay *φ* as a function of the cavity separation *r*. The laser modulation frequency is set at *ω*′* *= 56.51 kHz, which is close to the resonant frequency *ω*_0_ (* *= 43.79 kHz) of the cantilever system, so that a measurable ac signal is obtained with a low laser intensity (~1 mW). The values of *A* are obtained in two ways. The red circles are obtained by integrating the measured power spectrum *|z*(*ω*)*|*^2^ and the solid lines are obtained using DM-AFM with a lock-in amplifier. The measurements are conducted when the glass sphere is either approaching (with *r *< 0) or receding (with *r* > 0) from the lower surface of the cavity at a constant speed of *V *= 100 nm/s. (**a**) and (**b**) An infrared laser beam of wavelength *λ *= 1550 nm is used in a cavity formed between the gold-coated glass sphere and a plain quartz plate that does not generate any resonance effect. (**c**) and (**d**) An alignment red laser beam of wavelength λ* *= 635 nm is used in the plasmonic resonant cavity (for *λ *= 1550 nm) with the gold disk diameter *d* varying from 300 nm to 750 nm. (**e**) and (**f**) An infrared laser beam of wavelength *λ *= 1550 nm is used in the plasmonic resonant cavity with *d* varying from 300 nm to 750 nm. (**g**) and (**h**) Magnified plots of the amplitude *A* and phase delay *φ* shown in (**e**) and (**f**) in the near-filed region with *d* varying from 300 nm to 750 nm.

**Figure 5:**
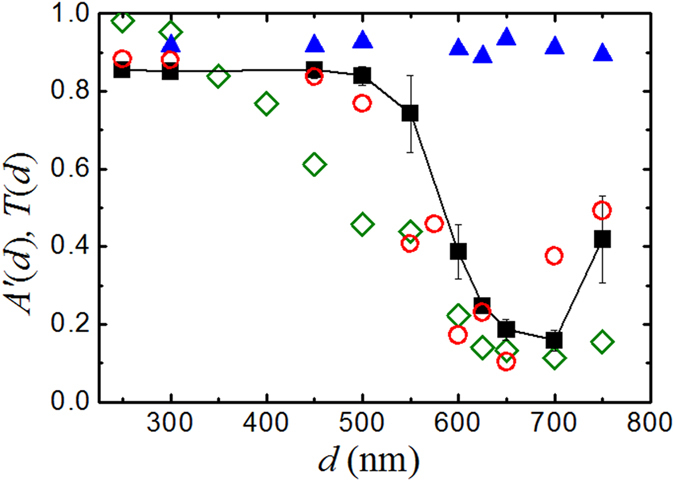
Comparison between the measured transmission coefficient *T*(*d*) (red circles) as a function of *d* for the infrared light of wavelength *λ *= 1550 nm and the normalized displacement amplitude *A*′_*1550*_(*d*) (black solid squares). The solid line is drawn to guide the eye. The error bars show the standard deviation of the measurements. Green diamonds are obtained from the numerical calculation of the transmission coefficient. Also shown is the normalized displacement amplitude *A*′_*635*_(***d***) (blue solid triangles) obtained in the same plasmonic resonant cavities but with the infrared laser light being replaced by an alignment red laser beam of wavelength *λ *= 635 nm.

**Figure 6:**
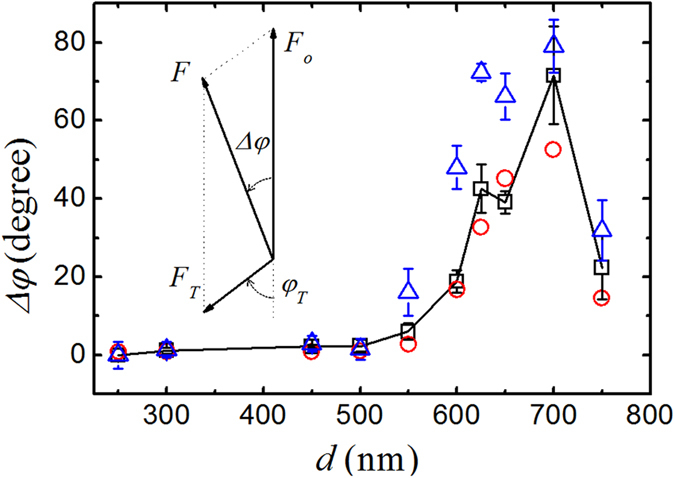
Measured phase shift Δ*φ *= *φ*(*d*) – *φ*(*d *= 250 nm) (relative to the non-resonant reference value obtained with *d *= 250 nm) as a function of gold disk diameter *d*. The measurements are made in the far field with *r* *=* 5 μm (black squares) and in the near field with *r* *=* 30 nm (blue triangles). The solid line is drawn through the black squares to guide the eye. The error bars show the standard deviation of the measurements. Inset depicts a vector sum of the total force *F* resulting from the two forces *F*_*o*_ and *F*_*T*_ with a phase delay *φ*_*T*_. Red circles are the calculated values of Δ*φ* using Eq. (5) with a constant value of *α* *=* (*F*_*T*_/*F*_*o*_)*T*(*d*) = 0.17.

**Figure 7:**
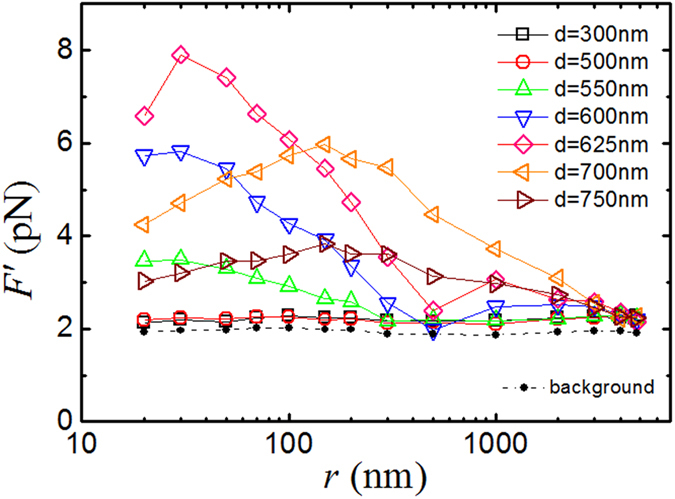
Measured optical force *F*′ normalized by transmission as a function of cavity separation *r*. The measurements are made in the plasmonic resonant cavity with gold disk diameter *d* varying from 300 nm to 750 nm. The solid lines are drawn to guide the eye. The black solid circles (with a dashed line) show the measured background force acting on the AFM cantilever beam.

The value of the displacement amplitude *A* is obtained in two different ways. The first is to integrate the sharp spike at the driving frequency *ω*′ = 56.51 kHz in the measured *|z*(*ω*)*|*^2^ under the PSD mode [see Eq. (3)] and the results are shown by the red circles in Fig. 4(a). The second way is to use the dynamic mode AFM with a lock-in amplifier and the results are shown by the solid lines in Fig. 4(a). In the latter case, the measurements are conducted when the glass sphere is either approaching (with *r* < 0) or receding (with *r* > 0) from the lower surface of the cavity at a constant speed of *V* = 100 nm/s. It is seen from Fig. 4(a) that the values of *A* obtained in the two methods are very close to each other; the difference between the two sets of data is less than 3%. The two methods complement each other. With the lock-in amplifier, one can measure the continuous variations of the amplitude *A* and phase delay *φ* as a function cavity separation *r*. From the measured *|z*(*ω*)*|*^2^, on the other hand, one can obtain the value of other parameters, such as *m*, *ω*_0_ and *ξ*, in addition to *A* and calculate the optical force *F*_*o*_ using Eq. (3).

## Additional Information

**How to cite this article**: Guan, D. *et al.* Direct Measurement of Optical Force Induced by Near-Field Plasmonic Cavity Using Dynamic Mode AFM. *Sci. Rep.* **5**, 16216; doi: 10.1038/srep16216 (2015).

## Figures and Tables

**Table 1:** Measured physical dimension of the gold disks on the quartz substrate for different nominal (designed) values of disk diameter *d.*

*d* (nm)	250	300	450	500	550	600	625	650	700	750
*đ* (nm)	170	242.5	392.5	465	495	545	567.5	610	630	695
*d*_up_ (nm)	130	205	315	400	430	460	470	530	550	610
*d*_lo_ (nm)	210	280	470	530	560	630	665	690	710	780
*τ* (nm)	16	16	15.5	15.5	15.5	15.5	15.5	15.5	15.5	15.5
*F*_*o*_*/F*_*T*_	5.031	5.004	5.032	4.938	4.367	2.272	1.453	1.092	0.929	2.459
*F*_*o*_*/F*	0.924	0.927	0.930	0.929	0.930	0.913	1.039	0.908	1.487	0.945

From the AFM image shown in Fig. 1(c), we find the actual shape of the gold disk is like a thin truncated circular cone with upper diameter *d*_up_, lower diameter *d*_lo_ and thickness *τ*. The mean diameter of the gold disks is defined as *đ* *=* *(d*_up_ + *d*_lo_)/2, which is smaller than the nominal value of *d* as listed blow, whereas the measured period of the nano-disk array is found to be accurate as the designed value of *d* + 200 nm. The calculated values of *F*_*o*_*/F*_*T*_ and *F*_*o*_*/F* in the far field using Eq. (5) are also listed for different values of *d*.
